# Generation and Characterization of iPS Cells Derived from APECED Patients for Gene Correction

**DOI:** 10.3389/fendo.2022.794327

**Published:** 2022-04-01

**Authors:** Eira Karvonen, Kai J. E. Krohn, Annamari Ranki, Annika Hau

**Affiliations:** ^1^Department of Dermatology and Allergology, University of Helsinki and Helsinki University Hospital, Helsinki, Finland; ^2^Clinical Research Institute Helsinki University Central Hospital (HUCH), Helsinki, Finland

**Keywords:** iPSC, pluripotency, autoimmunity, APECED, cell therapy

## Abstract

APECED (Autoimmune-Polyendocrinopathy-Candidiasis-Ectodermal-Dystrophy) is a severe and incurable multiorgan autoimmune disease caused by mutations in the *AIRE* (autoimmune regulator) gene. Without functional AIRE, the development of central and peripheral immune tolerance is severely impaired allowing the accumulation of autoreactive immune cells in the periphery. This leads to multiple endocrine and non-endocrine autoimmune disorders and mucocutaneous candidiasis in APECED patients. Recent studies have suggested that AIRE also has novel functions in stem cells and contributes to the regulatory network of pluripotency. In preparation of therapeutic gene correction, we generated and assessed patient blood cell-derived iPSCs, potentially suitable for cell therapy in APECED. Here, we describe APECED-patient derived iPSCs’s properties, expression of AIRE as well as classical stem cell markers by qPCR and immunocytochemistry. We further generated self-aggregated EBs of the iPSCs. We show that APECED patient-derived iPSCs and EBs do not have any major proliferative or apoptotic defects and that they express all the classical pluripotency markers similarly to healthy person iPSCs. The results suggest that the common AIRE R257X truncation mutation does not affect stem cell properties and that APECED iPSCs can be propagated *in vitro* and used for subsequent gene-correction. This first study on APECED patient-derived iPSCs validates their pluripotency and confirms their ability for differentiation and potential therapeutic use.

## Introduction

Autoimmune-Polyendocrinopathy-Candidiasis-Ectodermal-Dystrophy (APECED), also known as Autoimmune Polyendocrine Syndrome type 1 (APS-1), is a rare and severe multiorgan autoimmune disease caused by mutations in Autoimmune Regulator (*AIRE*) (1, 2). APECED (OMIM 240300) is characterized by a triad of manifestations including Addison disease (AD), hypoparathyroidism, and chronic mucocutaneous candidiasis (CMC) (3, 4). Additionally, considerable phenotypic variation of the disease is common including symptoms of varying severity of hypogonadism, hypothyroidism, hypophysitis, type 1 diabetes, vitiligo, and alopecia (5, 6). This syndrome is usually diagnosed during early childhood or adolescence, and it develops progressively to varying manifestations with increasing severity (7). APECED patient mortality is significantly increased in all age groups due to adrenal, and hypocalcemic crises, and oral squamous cell cancer, autoimmune hepatitis, pneumonitis, and nephritis (6, 8) as well as alcohol-, and accident-related deaths (9). Additionally, a significantly higher mortality is found to correlate with patients manifesting multiple endocrine symptoms (9). At present, there is no cure for APECED, and management consists of life-long hormone replacement therapy, and treatment of chronic candidiasis (6, 8, 10).

APECED is a monogenic disease with the most common AIRE mutation being the so-called “Finn-major” R257X mutation (11). This nonsense mutation results in the carboxyterminal truncation of AIRE leaving it non-functional and altering its subcellular localization (12). AIRE’s classical function within the immune system is to act as a transcription-factor like protein in helping release stalled RNA polymerase II and assisting in translation elongation (13). Functional AIRE is essential for the proper development of central and peripheral tolerance (14, 15). Its expression is tightly regulated in tissues of the immune system such as the thymus and lymph nodes as well in peripheral lymphoid tissues such as within the bone marrow (16, 17). The establishment of immune self-tolerance takes place in the thymus, where AIRE orchestrates the promiscuous gene expression of over 3200 tissue-specific self-antigens (TSAs) in medullary thymic epithelial cells (mTECs) (18–20). Defective AIRE leads to impaired expression and decreased surface display of TSAs to naïve T cells by mTECs. This loss of TSA presentation allows the survival of autoreactive T cells and their escape into the periphery from which they attack target tissues and molecules causing autoimmune manifestations (18). Additionally, also B-cell function and tolerance is abrogated as patients already harbor hundreds of neutralizing autoantibodies notably against a wide group of cytokines (21).

Recently, novel findings have shown *AIRE* to be expressed in stem cells, suggesting it has unforeseen functions outside the immune system. Germ cell progenitors have been found to express *AIRE* and its mutations were shown to cause fertility defects both in mice and in APECED patients (22–24). In addition, *AIRE* is highly active in undifferentiated embryonic stem cells (ES cells) and embryos with its expression decreasing during ES cell differentiation (25, 26). In mouse ES cells, knockdown of *Aire* reduces expression of the classical stem cell markers Oct4 and Nanog diminishing ES cell self-renewal potential (26). Moreover, Aire has been shown to activate another pluripotency inducer LIN28 thus suggesting an active contribution in the regulation and maintenance of pluripotency (25).

The development of gene-correction and iPSC technology has opened up opportunities for precision medicine and therapeutic avenues for monogenic diseases such as APECED. Recently, thymic precursor cells generated from human stem cells were shown to form functional thymic epithelial cells capable of supporting normal T cell development *in vivo* (27). AIRE expression has also been detected in human epidermal keratinocytes (28) that bear significantly similar biological attributes to thymic epithelial cells (13). Moreover, in the absence of a thymus, AIRE + keratinocytes have been shown to restore and support the production of mature, functional T cells concurrently promoting the culling of autoreactive cells (29). We propose that lentiviral re-introduction of a functional *AIRE* gene into patient-derived iPSCs, subsequent differentiation into suitable cell type(s) and re-introduction into affected individuals could elicit a curative effect. However, very little is known about the role of stem cells in APECED or their potential in therapeutic approaches and so far no publications have examined patient-derived iPSCs. Thus here, we generated to our knowledge the first APECED-patient derived iPSCs of two patients harboring the *AIRE* R257X mutation. We investigated the effects of the R257X mutation on iPSC proliferation, apoptosis, stem cell marker expression as well as well as the capability to differentiate normally into embryoid bodies (EBs) (30).

## Materials and Methods

This study was approved by Helsinki and Uusimaa Hospital District (HUS) Ethics Committee of Medicine (HUS/1127/2016) and all participating patients provided written informed consent.

### Cell Culture and iPSC Generation

For iPSC induction two adolescent female APECED patients with confirmed homozygous AIRE R257X mutations donated peripheral blood mononuclear cells that were then induced into iPSCs using the CytoTune-iPS 2.1 Sendai Reprogramming Kit (Invitrogen, Thermo Scientific) by Biomedicum Stem Cell Core (BSCC) (Helsinki, Finland). From both donors’ inductions approximately 10 iPSC clones were selected and propagated, with one clone per donor used for experiments (termed 137.2. and 138.6). The healthy control cell line HEL24.3 was similarly generated from an age-matched healthy individual at BSCC. After induction, all cell lines were assessed by the BSCC using multiple assays including immunohistochemistry and qPCR (for expression of OCT4, SSEA4, TRA-1-60) and RT-PCR to assess for the removal of transgene vectors (available upon justified request). The results were that all iPSC lines express endogenous pluripotent stem cell surface markers and Sendai virus vectors were absent from all lines thus meeting the common criteria for iPSC. HEL24.3 cells were induced from a commercial fibroblast cell line CCD1112Sk (ATCC) obtained from the foreskin of a Caucasian neonatal male. This cell line has been previously characterized in (31) and has been submitted to the Human Pluripotent Stem Cell Registry using the identifier UHi006-A (https://hpscreg.eu/cell-line/UHi006-A). Patient iPS cell lines 137.2 and 138.6 were karyotyped using G-band analysis (300-400 bands/haploid chromosome number according to ISCN 2020 guidelines (32) with 20 mitoses analyzed per cell line. The karyotyping was performed and analyzed by the accredited HUSLAB laboratory, HUS Diagnostic Center, Helsinki, Finland (https://huslab.fi).

The APECED patient -derived iPS cells were confirmed to contain the homozygous R25X mutations by sequencing at the HUSLAB laboratory, HUS Diagnostic Center, Helsinki, Finland. Relevant clinical and demographical information of the donors at time of donation are compiled in **Table 1**.

**Table 1:** Relevant clinical and demographical information of the donors at time of blood/tissue donation for the generation iPSC.

iPS cell line	Age/gender of donor	APECED disease components	AIRE mutation
137	17/female	CMC, HPT, AD	homozygote R257X (c.769 C>T)
138	15/female	CMC, HPT, AD, TIN	homozygote R257X (c.769 C>T)
HEL24.3	neonate/male	N/A	none

CMC, chronic mucocutaneous candidiasis; HPT, hypoparathyroidism; AD, Addison’s disease; TIN, tubulo-interstitial nephritis.

All iPS cells were cultured on Matrigel (Corning) -coated culture dishes using Essential 8 and Essential 8 Flex (Gibco) -media and passaged using 0,5 mM EDTA PBS (UltraPure 0.5M EDTA, Gibco; Phosphate Buffered Saline (1X) without Calcium and Magnesium, Lonza). All cells were kept in a humidified incubator at +37°C with 5% CO_2_.

### Generation of EBs

Self-aggregated EB suspensions were generated using a protocol modified from (33) and (34). On day 0, 80% confluent 6 cm iPSC culture dishes were rinsed with 0,5 mM EDTA, and then incubated 4 minutes in 0,5 mM EDTA. EDTA was aspirated, and colonies were harvested into a 6-well on Ultra Low Attachment plate (Corning Costar) with a 1:1 mix of Essential 8 media and DMEM (Dulbecco’s Modified Eagle Medium, Lonza) with 20% heat-inactivated FBS (Fetal Bovine Serum, Gibco) + 1% GlutaMAX (Gibco) and supplemented with 1x RevitaCell (Gibco). The cells were cultured in a humidified incubator at +37°C with 5% CO_2_. Next day (d1), media was replaced with EB growth media consisting of a 1:1 mix of DMEM + 10% FBS + 1% GlutaMAX and Essential 8 media. On days 2-4, 50-75% of the media was replaced daily. From day 5 onwards, 50-75% of the media was replaced every other weekday.

### RT-qPCR

The iPS cell cultures and EBs harvested on days 3, 7 and 30 were lysed with QIAzol (Qiagen) and total RNA was isolated using a chloroform, isopropanol, and ethanol purification according to Qiagen’s ‘Lysis and Homogenization of Fatty Tissues Using the Tissueruptor’ protocol followed by RNA cleanup using a RNeasy Mini Kit (Qiagen). Equal amounts of RNA per experiment were reverse-transcribed using High-Capacity cDNA Reverse Transcription Kit (Applied Biosystems) following manufacturer’s protocol. Expression levels were detected using iQ SYBR Green Supermix (Bio-Rad) and run in a LightCycler 480 II (Roche) with conditions: 1x 95°C 5 min; 40x (95°C 18s, 57°C 20s, 72°C 20s) using primers listed in **Supplementary Table 1**. Relative fold change of mRNAs were analyzed using the -2^-ΔΔCt^ method (35), with GAPDH1 used as a housekeeping gene.

### Immunocytochemistry

For cytospin preparates, iPSCs were detached using TrypLE Express (Gibco), resuspended into cold PBS and cytocentrifuged onto glass slides. The slides were fixed with 10% formalin for 10 min at RT. Samples of EBs were collected on days 3, 7 and 30 of culturing and fixed with 10% formalin for 20 min at RT. The EB samples were then dehydrated through increasing concentration of EtOH and lastly xylene, embedded in paraffin and sectioned onto glass slides. The FFPE EB samples were first deparaffinized through a rehydration series of xylene and decreasing concentrates of EtOH. The EB and iPSC samples were heated in citrate buffer (pH 6.2) for antigen unmasking, washed with 0.05% Tween20 (Sigma Aldrich) in PBS and blocked with 2.5% normal horse serum for 30 min at RT. The samples were then incubated with primary antibodies (listed in **Supplementary Table 2**), diluted into 1% BSA (Sigma Aldrich) in PBS) o/n at +4°C, except for Ki-67 in iPSCs which was incubated 1h at RT.

An ImmPRESS Duet Double Staining Polymer Kit (Vector Laboratories) was used to demonstrate colocalization of AIRE + OCT4/NANOG. After primary antibody incubation, the samples were incubated with the secondary antibody for 30 min at RT and stained with ImmPACT DAB EqV substrate (Vector Laboratories, RRID: AB_2336521) and Permanent HRP Green Kit (Nordic BioSite) at RT.

For Ki-67 immunostaining, the secondary antibody Vector Universal ImmPRESS kit (Vector Laboratories, RRID:AB_2336534) and Vector NovaRED Substrate Kit, Peroxidase (HRP) (Vector Laboratories, RRID:AB_2336845) were used.

Apoptosis was quantified using the ApopTag Peroxidase *In Situ* Apoptosis Detection Kit (Millipore, RRID:AB_2661855) according to the manufacturer’s protocol. The color was developed with Vector NovaRED Substrate Kit, Peroxidase (HRP) (Vector Laboratories, RRID:AB_2336845) at RT.

Lastly, samples were counterstained with Meyer hematoxylin, and after dehydration, overlaid with Pertex (Histolab) and covered with coverglass. Samples were imaged with a Leica DMLB microscope. The images were captured with a MicroPublisher RTV 5.0 camera (QImaging) using QCapture Pro 6.0 -software. Immunocytochemistry stainings were scored in blind by two individuals estimating the amount of positive cells on a scale of 0-33%, 34-66%. 67-99%, and > 99% cells per sample. Representative images of scorings are shown.

All statistical analyses were performed in IBM^®^ SPSS^®^ Statistics version 25. Results were analyzed using one-way ANOVA and Dunnett’s t-tests.

## Results

### APECED Patient-Derived iPSCs Do Not Have Any Major Proliferative Defects

APECED patient-derived PBMCs were induced to iPS cells with the widely used CytoTune system. During and upon induction, the pluripotent stem cells displayed normal induction characteristics, morphology and formed iPSC colonies at expected efficiency (data not shown). Approximately 10 clones per patient were generated and two clones per patient selected for detailed experimental analysis. After passaging the APECED iPSC cells for 40+ passages, no discernible differences between the clones from any single patient have arisen. All clones exhibit a normal stem cell -associated morphology and display typical pluripotency characteristics: the cells are small and form tightly packed, round colonies with distinct borders and a high nucleus-to-cytosol ratio (30). Therefore, only one clone per patient was selected for detailed studies herein. Additionally, these two clones termed 137.2 and 138.6 were analyzed by standard Giemsa-band staining in a karyotyping assay showing both patient cell lines to have normal 46, XX karyotypes devoid of any structural or numerical chromosome abnormalities (data not shown, images available upon justified request).

**Figure 1:**
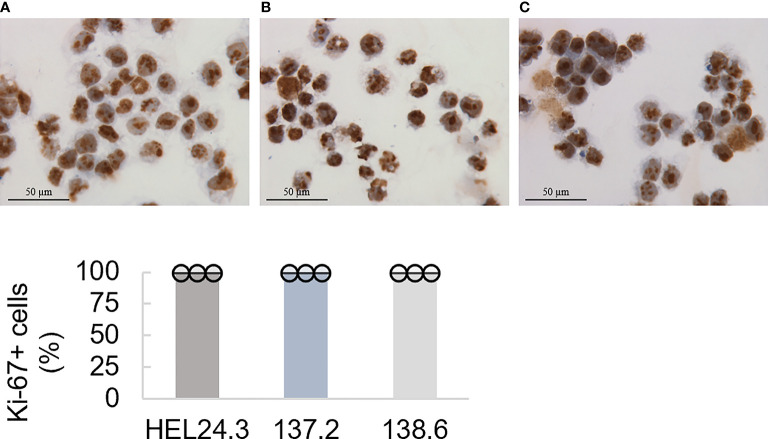
Proliferation capacity of APECED patient-derived iPSCs. Top panel: The healthy control iPSCs HEL24.3 **(A)** and APECED iPSCs 137.2 **(B)** and 138.6 **(C)** display similar Ki-67 positivity. Bottom graph: Quantitation of proliferation (Ki-67+) in the iPSCs reveals they are similar irrespective of *AIRE* mutation. Dots represent three individually performed replicates, with error bars representing SD within a replicate. Bar graphs are the mean of three replicates, with error bars representing SEM of the replicates. No difference was observed between the cell lines.

As these cells had no discernible phenotypic or morphologic differences to the healthy control clone HEL24.3, we wanted to study their proliferative properties in more detail. Using immunocytochemistry, we analyzed the expression of nuclear protein Ki-67 (36) which is a widely used marker for proliferation. We found that APECED iPSCs display comparable proliferative capacity to healthy control iPSCs with no quantifiable difference in Ki-67 positivity (**Figure 1**), showing that APECED patient-derived iPSC do not have any proliferative defects.

### APECED iPSCs Display Similar Stem Cell Marker Expression as Healthy Control iPSCs

As AIRE-deficient iPSCs have not previously been developed or characterized, we wanted to validate their pluripotency potential by studying whether these cells express the core pluripotency genes *OCT4*, *SOX2* and *NANOG* (37) as well as stem cell defining genes *TDGF1*, *p53*, *MYC*, *CD95/Fas* and *LIN28* (38–40).

**Figure 2:**
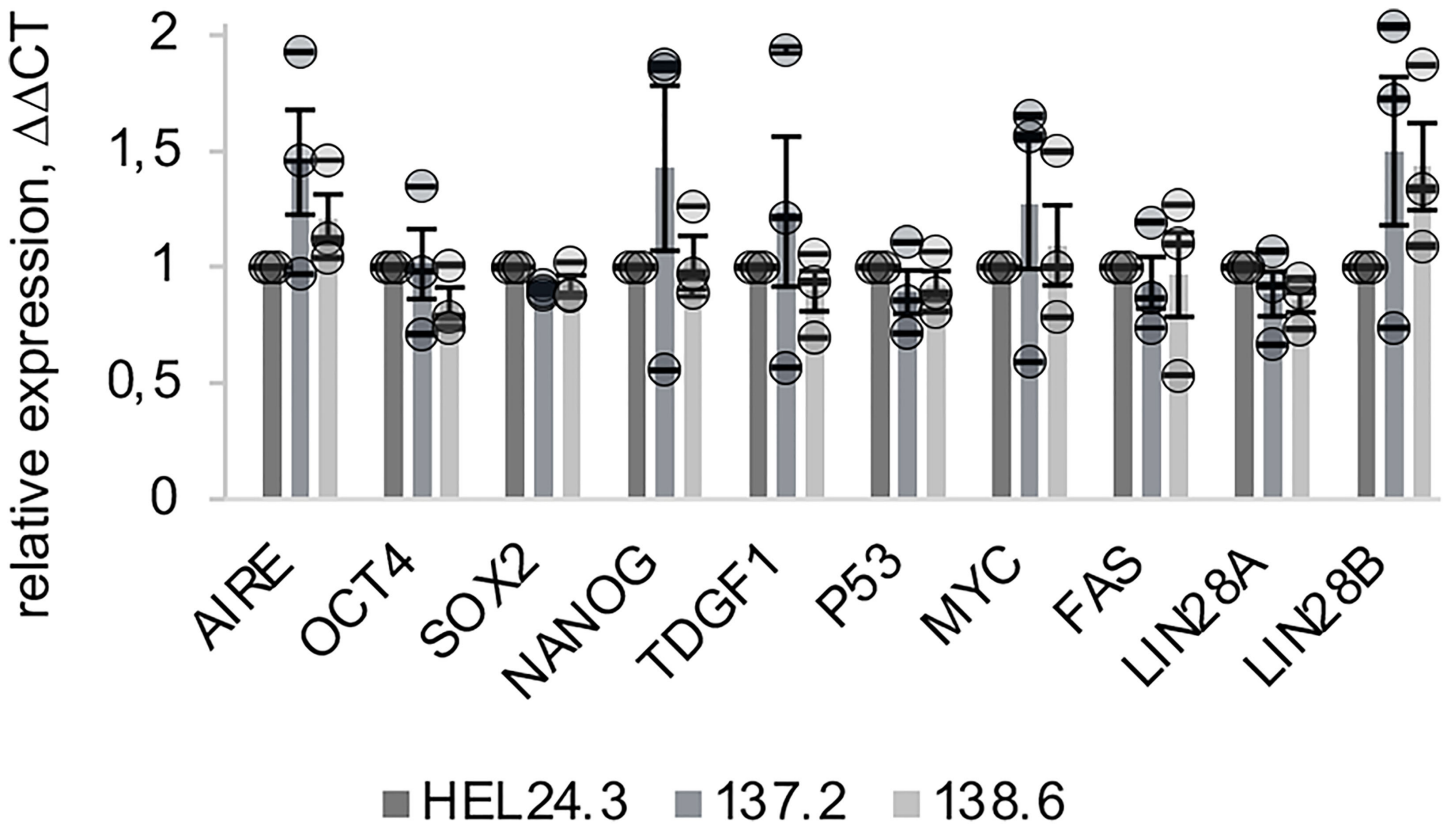
Expression of AIRE and stemness-associated genes does not differ in APECED patient -derived iPSCs compared to healthy person-derived iPS cells. The relative mRNA expression of AIRE and common stem cell associated markers in APECED iPSCs (137.2 & 138.6) and a healthy control (HEL24.3) iPSCs. Expression levels presented as a mean of ΔΔCt fold change (columns) of three independently analyzed biological replicates (shown as circles), normalized to HEL24.3. Error bars of circles represent SD within a replicate. Error bars of columns represent SEM of the three replicates. No statistically significant difference was observed between the cell lines (p > 0.05).

Firstly, we examined by quantative real time PCR (qPCR) the mRNA expression of these markers in iPSCs. Expression of *p53*, *Myc* and *CD95/Fas* mRNAs was similar in both AIRE wild type and AIRE R257X iPSCs suggesting that loss of functional AIRE does not affect the aforementioned factors. Additionally, the expression of *LIN28A* and *LIN28B* were assessed and *LIN28B* showed an increase, though it was not statistically significant (**Figure 2**).

**Figure 3:**
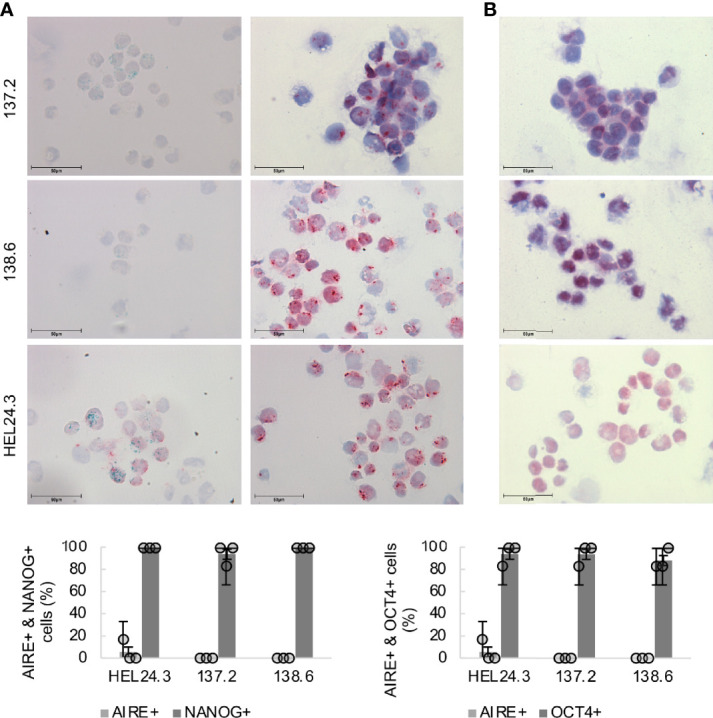
Subcellular localization of AIRE and stemness-associated proteins does not differ in APECED patient -derived iPSCs compared to healthy person-derived iPS cells. Top panel **(A)**: AIRE (green) and NANOG (red) expression in iPSCs, **(B)**: OCT4 (red) in iPSCs. Mayer’s hematoxylin (blue) used as nuclear counterstain. Bottom graph: Quantitation of AIRE+, NANOG+ and OCT4+ in healthy control iPSCs HEL24.3 and APECED iPSCs 137.2 and 138.6. Circles represent three individually performed replicates, with error bars representing SD within a replicate. The bar graphs represent the mean of the three replicates, with error bars representing SEM of the replicates. No statistically significant difference was observed between the cell lines (p > 0.05).

Next, protein expression and subcellular localization of AIRE, NANOG and OCT4 were examined using ICC on cytospin samples of APECED patient and healthy control iPSCs. The results of the AIRE and NANOG immunostaining are presented in **Figure 3**. No observable differences were found in the core stem cell markers’ expression as the percentage of NANOG or OCT4 positive cells was comparable in the APECED iPSCs to the healthy control iPSCs. Next, we wanted to assess AIRE expression using an antibody raised against AIRE’s N-terminal region. This antibody recognizes both the wild type as well as the truncated R257X mutant AIRE protein (41) and we found that both wild type HEL24.3 cells as well as patient iPSCs clones 137.2, and 138.6 expressed AIRE, however at very low levels with expression detected only in a few cells. Both the truncated AIRE in patient cells as well as the wild type AIRE in healthy person cell line were detected as a punctate pattern mostly in the cell nuclei but also in the cytoplasm as has been previously reported for AIRE (42, 43). Colocalization of AIRE and NANOG was detected in a few cells of healthy control HEL24.3 cell line, but not in APECED patient cell lines.

### AIRE Deficient EBs Show No Gross Proliferative Defects

The ability to form EBs defines all pluripotent cells including iPSCs and they resemble the blastocyst phase of early embryos in their gene expression and epigenetic landscape (44). EBs also represent the onset of differentiation (45, 46) and thus we wanted to examine in detail whether mutated *AIRE* alters patient iPSC’s pluripotency and early differentiation capacity. We generated EBs from patient and healthy control cells using the self-aggregation method (33, 34) observing the EB organoids for up to 100 days.

**Figure 4:**
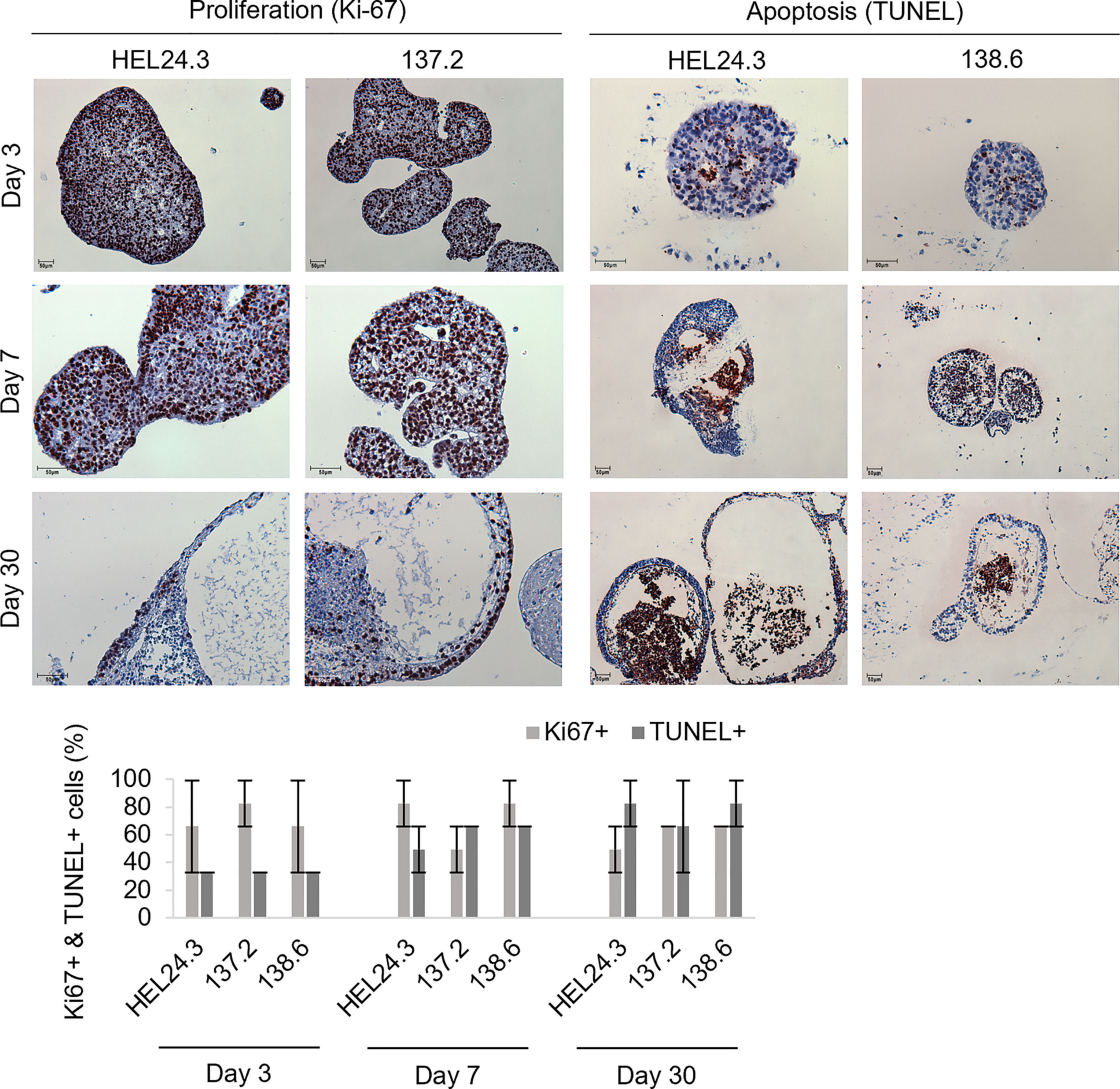
Proliferation in APECED iPS-derived EBs is seen along the cortical layers and apoptosis within the medulla creating a lumen. Top panel: Proliferative (Ki-67, brown) and apoptotic (TUNEL, brown) cells in EBs generated from iPS cells of one healthy control HEL24.3 (first column and third column), and in two APECED iPS cell lines 137.2 (second column) and 138.6 (fourth column) as detected by Ki-67 and TUNEL immunocytochemistry, respectively. The FFPE samples were collected on days 3, 7 and 30 after generation of the EBs. Mayer’s hematoxylin (blue) as nuclear counterstain. Bottom graph: Quantitation of proliferation (Ki-67+) and apoptosis (TUNEL+) in the EBs reveals they are similar irrespective of AIRE mutation. Error bars represent SD.

Early EBs were typically dense, solid spheroids but later the majority became cyst-like structures with projections emerging from the spheric central mass. To quantify the proliferative and apoptotic indexes of these EBs we chose to analyze their Ki-67 (36) and TUNEL activity (47). For this, we generated EBs from healthy HEL24.3 and APECED patient 137.2 and 138.6 clones, and harvested samples on days 3, 7, and 30. As shown in **Figure 4**, no discernible differences in proliferative nor apoptotic capacity was noted in APECED patient EBs. Indeed, all EBs had similar kinetics and distribution of apoptotic to proliferative cells. Furthermore, in all young EBs, the proliferative and apoptotic cells were typically evenly dispersed throughout the organoids. After day 7, the localization of proliferative and apoptotic cells became more dualistic, as Ki-67 was found especially on the cortical part of EBs, whereas apoptosis by TUNEL positivity was most prominent in the medullary regions (**Figure 4**). This reflects the classical event of clearance of the EB luminal cavity through caspase-dependent apoptosis (44).

### AIRE and Stem Cell Marker Expression in APECED EBs is Similar to Healthy EBs

**Figure 5:**
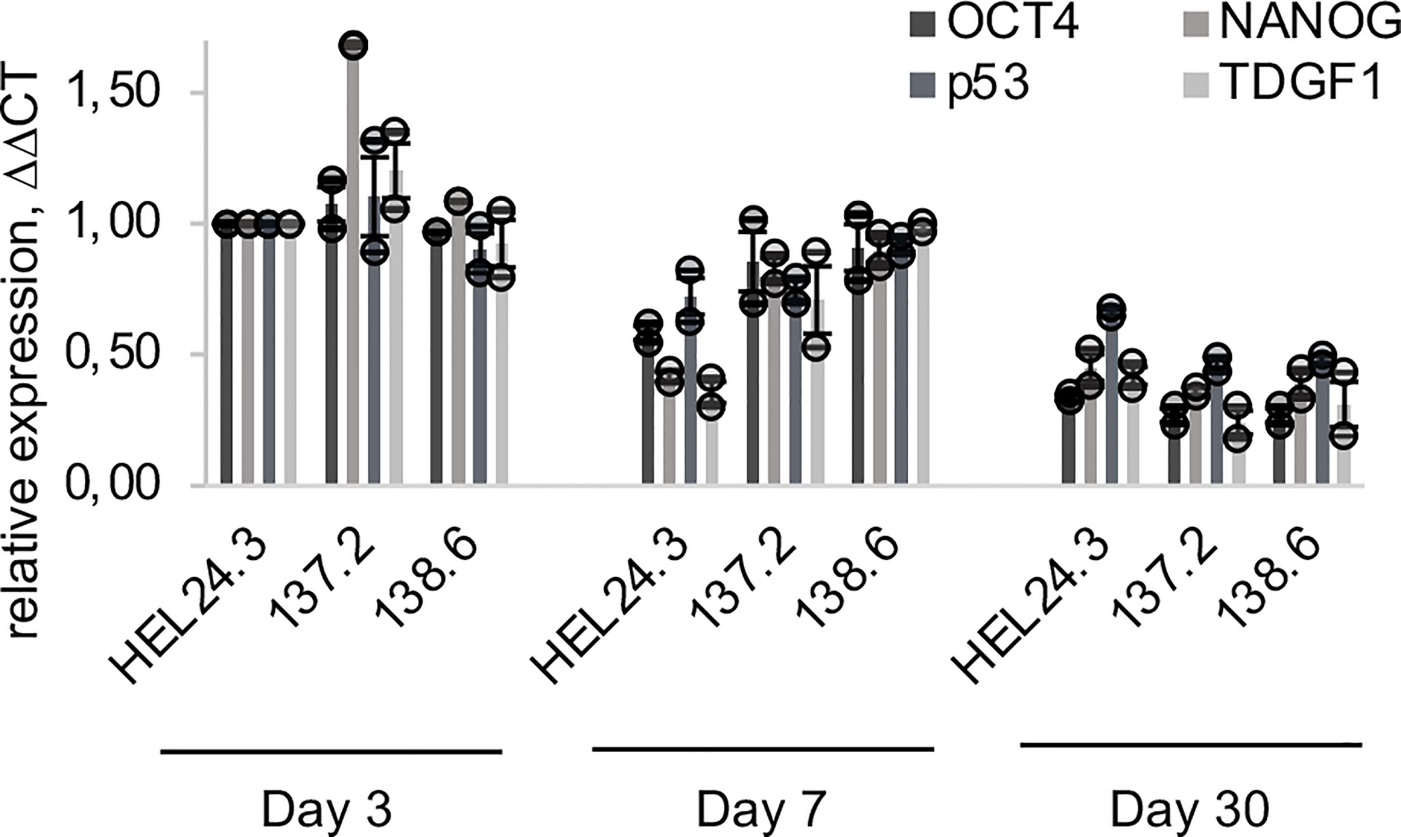
qPCR quantification of *OCT4, NANOG*, *p53* and *TDGF1* expression in APECED and healthy person iPS-derived EBs. The relative mRNA expression in APECED EBs (137.2 and 138.6) and a healthy control (HEL24.3) EBs presented as a mean of ΔΔCt fold change of 2 independently analyzed biological replicates (shown as circles), normalized to the healthy control HEL24.3 day 3 sample. Error bars of the circles represent SD within a replicate. Error bars of the bars represent SEM of the two replicates.

Next, we set out to quantify the mRNA expression of the core stem cell factors by qPCR in day 3, 7, and 30 aged EBs. The results showed that *OCT4, NANOG, p53* and *TDGF1* mRNAs have similar kinetics and their expression decreased towards day 30 (**Figure 5**). This is consistent with published data where “ageing” of EBs correlates with decreased pluripotency marker expression (44).

**Figure 6:**
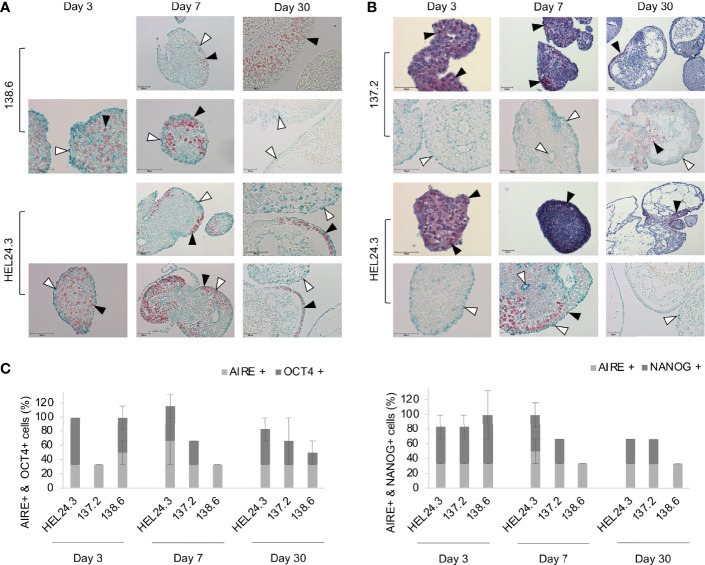
AIRE mutation does not affect the spatial localization of AIRE, OCT4, or NANOG proteins in EBs. Panel **(A)** AIRE (green staining, indicated with white arrow heads) and OCT4 (red staining, indicated with black arrow heads) protein expression in EBs generated from iPS cells of APECED patient 138.6 and healthy control HEL24.3 on day 3, 7 and 30 after the generation of EBs. Panel **(A)** images are without Mayer’s hematoxylin counterstaining. Panel **(B)** AIRE (green staining, indicated with white arrow heads) and NANOG (red staining, indicated with black arrow heads) protein expression detected by immunocytochemistry in EBs generated from iPS cells of APECED patient 137.2 and healthy control HEL24.3. FFPE samples collected on days 3, 7 and 30 after the generation. Mayer’s hematoxylin (blue) used as a nuclear counterstain on images on the first and third rows. Images on second and fourth row are without Mayer’s hematoxylin. Scale bar equals 50 µm. Panel **(C)** Quantification of positive cells in AIRE + OCT4 and AIRE + NANOG immunostainings shown in panels **(A**, **B)**. Error bars represent SD.

We also set out to study stem cell marker protein localization within the EBs with immunocytochemistry. On day 3, EBs contained a marked number of OCT4 and NANOG-positive cells that were evenly dispersed throughout the individual EBs (**Figures 6A, B**), respectively). In day 7 and especially in day 30 EBs, the expression of NANOG and OCT4 became restricted to well defined areas of the outermost cortical layer. The number of positive cells and staining intensity of OCT4 and NANOG decreased as the EBs grew older. These data are in line with our qPCR data in **Figure 5** where both these factors’ mRNAs decrease similarly. As for AIRE, the most intense staining was detected in the cortical layers of the EBs though some AIRE was also detected in the medulla and AIRE and NANOG/OCT4 stained positive in distinct cells and areas of the EBs (**Figure 6**).

## Discussion

In this study we wanted to explore a novel aspect of AIRE’s potential role in induced pluripotent stem cells. We generated two iPSC lines from two female APECED patients and to our knowledge, this is the first study to generate and characterize APECED patient-derived iPSCs and to analyze the effects of their AIRE R257X truncation mutation. In addition to AIRE’s pivotal role in establishing immune tolerance, recent studies have implicated AIRE in the self-renewal of ES cells, as Aire^-/-^ ES cells have a significant impairment in their proliferation and organoid formation capacity. Additionally, another AIRE mutant was shown to induce mitotic defects during human reproduction and development (23, 26). Here we report initial data that APECED patient-derived iPSCs, harboring the AIRE R257X truncation mutation, have typical stem cell morphological features and display no impairment in proliferation as measured by Ki-67 positivity (**Figure 1**). The mutated AIRE protein was detectable by immunocytochemistry both in a nuclear (48) and a cytoplasmic, punctate pattern (49) as described before (42, 43). *AIRE* mRNA was detectable in very low quantities in iPSCs as shown by qPCR (**Figure 2**). Also, when we analyzed AIRE protein expression in iPSCs and EBs, only a small subset of cells stained positive. This expression pattern in pluripotent cells could reflect the stochastic and tightly controlled *AIRE* expression seen in thymic mTEC cells (18, 20). To our knowledge, there are no previous immunohistochemical analyses on AIRE protein abundance in pluripotent cells nor are there any assessments whether AIRE expression is limited only to a subset of stem cells or if it is more abundantly expressed. Based on our findings we propose that the functionally defective truncated AIRE does not seem disadvantageous to the overall proliferation capacity of iPSCs nor does it disrupt their ability to undergo the initial stages of differentiation as evidenced by EB formation.

Here, to validate the pluripotency of the APECED patient-derived iPSCs, expression of a set of stem cell factors and markers was assessed using qPCR. All iPSC clones independently of their AIRE mutation status expressed high levels of the pluripotency core genes, as well as stem cell associated markers *TDGF1* and *LIN28* (38) (**Figure 2**). This is in clear contradiction to previous data from animal models where Aire knockdown reduced the expression of Oct4 and Nanog in mouse ES (mES) cells thus decreasing their self-renewal capability (26). However, a possible explanation could be either iPSC heterogeneity (50) or that a full knockout of AIRE causes different biological outcomes compared to our R257X truncation mutant located in the SAND domain of AIRE. AIRE contains four major subdomains: the CARD/HSR, the SAND and two PHD finger- type zinc finger domains. The AIRE protein also contains four LXXLL domains that are found on coactivators of nuclear receptors (reviewed in (4)). Although the functions of these different domains of AIRE are established, the clinical outcomes of each individual AIRE mutation are still unknown. Currently over 100 AIRE mutation variants have been identified and only a subset have been exhaustively characterized for even their most fundamental clinical outcomes and associations. Also, as seen in APECED animal models, the manifestations of the AIRE mutation spectrum varies greatly depending on genetic background and species (4, 51). Thus it is entirely plausible, as our data suggest, that human AIRE-dependent pluripotency regulation could be more nuanced than in mouse models.

LIN28’s two paralogues LIN28A and LIN28B are one of the classical stem cell markers and they regulate a complex network including OCT4, NANOG and SOX2 (25, 52). Aire has previously been shown to support the self-renewal of mouse ES cells through the activation of Lin28 and loss of AIRE lead to decreased expression of Lin28 (25). However, in this study the mRNA expression of *LIN28A* in APECED patient-derived iPSCs was only slightly decreased compared to healthy control iPSCs. This suggests that activation of LIN28A in humans might not be AIRE-dependent. However, we cannot exclude the possibility that this is due to the limited amount of iPSC clones we studied (one per patient) or the inherent heterogeneity in gene expression among iPS cell lines (50, 53–55). Also, CD95/FAS, p53 and MYC had comparable expression patterns both in heathy control HEL24.3 and APECED patient iPSCs clones 137.2 and 138.6.

The ability to form EBs is a quintessential feature of pluripotent stem cells enabling their propagation for extended periods of time (44). Thus, we chose to generate EBs to further investigate the stemness and early developmental phase properties of APECED patient derived iPSCs and to examine whether APECED EBs differ in stemness or proliferation. Satisfyingly, we detected no discernible differences in APECED EBs compared to healthy control EBs. The expression of OCT4 and NANOG, the core markers of pluripotency (37), decreases during differentiation (**Figure 5**) as expected (38, 56). We did not find colocalization of AIRE and NANOG or AIRE and OCT4, as AIRE and NANOG/OCT4 protein immunostainings were found in distinct cells and areas of the EBs. In early EBs on day 3, OCT4 and NANOG expression was detected evenly throughout the organoids. Later the expression of NANOG and OCT4 became restricted to areas of the cortical layer and the number of positive cells decreased as the EBs grew older (**Figure 6**). This is in accordance with previous reports, as OCT4 expression is found in endoderm-like cells within the EB core and later during differentiation its expression is ultimately lost (44). Within early EBs, the proliferative Ki-67+ and apoptotic TUNEL+ cells were found evenly throughout the EBs but later their localization became almost mutually exclusive as Ki-67+ was found in the EB cortex and TUNEL+ on the edges and within the lumens. These results are in line with earlier reports showing young EBs as solid spheres which later evolve into cyst-shaped EBs with an external proliferative cell layer and internal apoptosis (57). No difference was detected between the healthy control and APECED EBs indicating that the R257X truncated AIRE does not seem to thwart normal proliferation of iPSCs.

Moreover, our results indicate that the most prevalent AIRE mutation (p.R257* located in the SAND domain) does not affect stem cell marker expression nor the pluripotency/EB formation of human iPSCs. This despite previous studies with Aire knockout mice have shown that both the mRNA and protein level of the core stem cell factors Oct4 and Nanog were decreased in Aire^-/-^ ES cells and that the Aire^-/-^ ES cells manifested impaired proliferation and colony formation (23, 26). One possible reason for these conflicting results might be that the truncated R257X AIRE in APECED patients could still be capable of performing some functions compared to the complete loss of AIRE in knockout animal models.

## Conclusions

This study provides the first insights into APECED patient derived iPSCs and the effect of the most common underlying, disease-causing mutation in AIRE. Together these initial results indicate that the R257X mutation of the APECED patients does not abrogate pluripotency nor cause defects in proliferation, apoptosis, or the ability of these iPSCs to form EBs. These data open the possibility of generating iPSCs from APECED patients and using lentiviral gene-delivery to restore AIRE expression in these cells. While we did not attempt to differentiate the iPS cells we however postulate that possible differentiation into thymic precursors (27) or the keratinocyte lineage (58) could allow for the re-introduction of healthy autologous cells into APECED patients. Currently, we are commencing the restoration of keratinocytes in a rat model of APECED (51) where we will use a lentiviral vector constructed by us to restore endogenous expression of AIRE.

## Data Availability Statement

The original contributions presented in the study are included in the article/**Supplementary Material**. Further inquiries can be directed to the corresponding author.

## Ethics Statement

The studies involving human participants were reviewed and approved by Helsinki and Uusimaa Hospital District (HUS) Ethics Committee of Medicine (HUS/1127/2016). Written informed consent to participate in this study was provided by the participants’ legal guardian/next of kin.

## Author Contributions

Conceptualization of the study was done by KK, AH, and AR. Data curation, validation, visualization, methodology, and writing of the original draft were done by EK and AH. Moreover, EK, AH, and AR did the formal analysis and investigation of the study. The funding acquisition and project administration were supervised by AR and KK. AR was also in charge of the resources. The whole study was supervised, reviewed, and edited by AH and AR. All authors contributed to the article and approved the submitted version.

## Funding

This work was supported by grants from the Academy of Finland (grant 309433), Finska Läkaresällskapet, and Helsinki University Hospital Research Funds grant TYH2020235.

## Conflict of Interest

The authors declare that the research was conducted in the absence of any commercial or financial relationships that could be construed as a potential conflict of interest.

## Publisher’s Note

All claims expressed in this article are solely those of the authors and do not necessarily represent those of their affiliated organizations, or those of the publisher, the editors and the reviewers. Any product that may be evaluated in this article, or claim that may be made by its manufacturer, is not guaranteed or endorsed by the publisher.

## Supplementary Material

Supplementary Table 1 Primers used in qPCR

Supplementary Table 2 Primary antibodies used for immunostaining. The monoclonal AIRE 6.1 antibody was produced in-house from a mouse hybridoma (41) and the clone was generated against a peptide comprising amino acids 1-206 of the N-terminus of the AIRE protein.
