# Supplementary material for: Generation and Characterization of iPS Cells Derived from APECED Patients for Gene Correction
Source: Front Endocrinol (Lausanne). 2022 Apr 1;13:794327. doi: 10.3389/fendo.2022.794327 (PMC9010864; doi:10.3389/fendo.2022.794327)
Supplement: Supplementary Table 1 — Primers used in qPCR [file Table_1.pdf]

Supplementary Table 1. Primers used in qPCR

Gene	Accession N#.	Forward Primer (5'-3')	Reverse Primer (5'-3')	product size (bp)
GAPDH1	NM_002046.5	CTCTGCTCCTCCTGTTCGAC	GCGCCCAATACGACCAAATC	121
AIRE	NM_000383.3	CCCAGCTCCACCAGAAGAATG	GGTGGACCTCTTACCTCCTCT	277
OCT4	NM_001285986.2	TTGGGCTCGAGAAGGATGTG	TCCTCTCGTTGTGCATAGTCG	91
SOX2	NM_003106.4	GCCCTGCAGTACAACTCCAT	TGCCCTGCTGCGAGTAGGA	85
NANOG	NM_001355281.2	CTCAGCCTCCAGCAGATGC	TAGATTTCATTCTCTGGTTCTGG	94
TDGF1	NM_001174136.2	TCAGAGATGACAGCATTTGGC	TTCAGGCAGCAGGTTCTGTTTA	118
p53	NM_000546.5	TGTGACTTGCACGTACTCCC	ACCATCGCTATCTGAGCAGC	199
MYC	NM_002467.5	CAGCGACTCTGAGGAGGAAC	GCTGGTGCATTTTCGGTTGT	286
FAS	NM_152871	CCCTGTCCTCCAGGTGAAAG	CCTTTCTGTGCTTTCTGCATGT	367
LIN28A	NM_024674.5	AGATCAAAAGGAGACAGGTGCT	AATAGCCCCCACCCATTGTG	256
LIN28B	NM_001004317.3	GCAGAAGATCACTCCGTTCCA	CCTTCCATGAATAGTTTGCTTTGGT	290
