# Supplementary material for: Generation and Characterization of iPS Cells Derived from APECED Patients for Gene Correction
Source: Front Endocrinol (Lausanne). 2022 Apr 1;13:794327. doi: 10.3389/fendo.2022.794327 (PMC9010864; doi:10.3389/fendo.2022.794327)
Supplement: Supplementary Table 2 — Primary antibodies used for immunostaining. The monoclonal AIRE 6.1 antibody was produced in-house from a mouse hybridoma (41) and the clone was generated against a peptide comprising amino acids 1-206 of the N-terminus of the AIRE protein. [file Table_2.pdf]

Supplementary table 2. Primary antibodies used for immunostaining.

The monoclonal AIRE 6.1 antibody was produced in-house from a mouse hybridoma (41) and the clone was generated against a peptide comprising amino acids 1-206 of the N-terminus of the AIRE protein.

**Antibody**	**Host species**	**Dilution**	**Manufacturer**	**Product code**	**RRID**
AIRE 6.1	Mouse	1:100	in-house	N/A	N/A
Oct-4A (C52G3)	Rabbit	1:1000	Cell Signaling Technology	2890	AB_2167725
Nanog (D73G4) XP®	Rabbit	1:100	Cell Signaling Technology	4903	AB_10559205
Ki-67 (8D5)	Mouse	1:400	Cell Signaling Technology	9449	AB_2797703
